# Influence of genetic variants in the susceptibility and outcome of influenza virus infection

**DOI:** 10.1186/cc14107

**Published:** 2015-03-16

**Authors:** J Sole-Violan, M López-Rodríguez, E Herrera-Ramos, J Ruiz-Hernández, J Horcajada, L Borderías, J Blanquer, J Ferrer, O Rajas, J Aspa, F Rodríguez de Castro, C Rodríguez-Gallego

**Affiliations:** 1 Hospital GC Dr Negrín, Las Palmas de Gran Canaria, Spain; 2 Hospital del Mar, Barcelona, Spain; 3 Hospital San Jorge, Huesca, Spain; 4 Hospital Clínico, Valencia, Spain; 5 Hospital de la Princesa, Madrid, Spain

## Introduction

The role of genetic variability in the susceptibility and outcome of influenza virus infection (IVI) remains largely unknown. We have previously demonstrated that variants at SFTPA2 influence the severity of H1N1pdm infection. We have now studied genetic variants at different genes, some of them previously associated with infections by influenza and/or other viruses. The purpose of this study was to analyze the role of genetic variants in the susceptibility and outcome of IVI.

## Methods

In total, 136 white Spanish patients developed IVI (80.3% of them by H1N1pdm virus). The general population group consisted of 1,466 unrelated healthy volunteers. Patients and controls were analyzed for different polymorphisms at 13 genes (FCGR2A, FCGR3A, FCGR3B, IL1RN, IL6, LTA, TIRAP, TLR1, TLR2, TLR3, TLR4, CCR5, IGHG2). IVI was detected in nasopharyngeal swabs using real-time PCR. The Hardy-Weinberg equilibrium was analyzed by Haploview v. 4.2. The comparisons of genotypes distribution based on susceptibility and severity were performed using the chi-squared test or Fisher's exact test when needed. The relationship between severity in hospitalized patients and genotypes was evaluated by binary logistic regression models.

## Results

No associations were found between the different genetic variants and susceptibility or severity of IVI. Variants at LTA, FCGR2A, IGHG2, TLR3 and CCR5, previously associated with severity of IVI were not replicated in our study.

## Conclusion

Our study does not suggest that polymorphisms at LTA, FCGR2A, IGHG2, TLR3 and CCR5 genes are associated with susceptibility or severity of IVI.

